# Assessing Cyber Risks of an INS Using the MITRE ATT&CK Framework

**DOI:** 10.3390/s22228745

**Published:** 2022-11-12

**Authors:** Aybars Oruc, Ahmed Amro, Vasileios Gkioulos

**Affiliations:** Department of Information Security and Communication Technology, Norwegian University of Science and Technology, 2815 Gjøvik, Norway

**Keywords:** maritime cyber security, risk assessment, INS, integrated navigation system, MITRE ATT&CK framework

## Abstract

Shipping performed by contemporary vessels is the backbone of global trade. Modern vessels are equipped with many computerized systems to enhance safety and operational efficiency. One such system developed is the integrated navigation system (INS), which combines information and functions for the bridge team onboard. An INS comprises many marine components involving cyber threats and vulnerabilities. This study aims to assess the cyber risks of such components. To this end, a methodology considering the MITRE ATT&CK framework, which provides adversarial tactics, techniques, and mitigation measures, was applied by modifying for cyber risks at sea. We assessed cyber risks of 25 components on the bridge by implementing the extended methodology in this study. As a result of the assessment, we found 1850 risks. We classified our results as 1805 low, 32 medium, 9 high, and 4 critical levels for 22 components. Three components did not include any cyber risks. Scientists, ship operators, and product developers could use the findings to protect navigation systems onboard from potential cyber threats and vulnerabilities.

## 1. Introduction

Over 80% of goods in international trade are carried by ships [1]. One of the most essential elements of maritime transportation is explicitly ships. In 2020, the worldwide merchant fleet grew by 3% and reached 99,800 ships of 100 gross tons and above [1]. Contemporary ships are equipped with computerized systems for different purposes, such as navigation, communication, propulsion, and cargo handling. The safety and operational efficiency of vessels are improved because of such systems. However, these systems are accompanied by growing cyber security concerns in the maritime industry because of experiencing cyber incidents and revealing research results.

The International Maritime Organization (IMO) is the responsible agency in the United Nations for the safety and security of shipping and the prevention of environmental pollution by ships [2]. Maritime cyber risk is defined by the IMO as “*a measure of the extent to which a technology asset is threatened by a potential circumstance or event, which may result in shipping-related operational, safety or security failures as a consequence of information or systems being corrupted, lost or compromised*” [3]. In 2017, the IMO issued a resolution to prevent maritime cyber risks [4]. As per the resolution in force, cyber risks must be assessed by ship operators and addressed in their approved Safety Management Systems (SMS). Moreover, they should make reference to the Ship Security Plan (SSP) as per the International Ship and Port Facility Security (ISPS) Code [5,6]. This requirement has been verified in the Document of Compliance (DOC) audits of ship operators since 2 January 2021.

This paper reveals the significance of cyber risks onboard vessels. We contributed to the literature by extending a methodology using the MITRE ATT&CK framework to assess the cyber risks of systems onboard ships. Moreover, the method was implemented to specifically assess the cyber risks of an INS in this study. A total of 1850 risks were classified as 1805 low, 32 medium, 9 high, and 4 critical levels. Given that no marine casualty (e.g., collision, explosion, injury, and oil spill) caused by cyber attacks was found in the literature, safety and environmental impacts of cyber risks are outside of the scope of this study.

We organised the remainder of the paper as follows. Section 2 gives a review of the related literature. In Section 3, the methodology is discussed and implemented for the cyber risks of an INS. Section 4 offers a summary and suggests additional research topics for further investigation. Consequently, in Appendix A, cyber risks of medium, high, and critical levels are listed.

## 2. Background

### 2.1. INS Concept

The IMO defines an INS as “A system in which the information from two or more navigation aids is combined in a symbiotic manner to provide an output that is superior to any one of the component aids” [7]. The INS aims to improve safe navigation by combining and integrating information and functions for the Officer of the Watch (OOW) in planning, monitoring, and controlling ship navigation [8]. An INS constitutes six navigational tasks as mandatory and optional, as follows:

- Route Monitoring: “*The navigational task of continuous surveillance of own ships position in relation to the pre-planned route and the waters*” [9].
- Route Planning: The task that provides procedures for voyage planning, route planning functions and data for the Electronic Chart Display and Information System (ECDIS), administering the route plan, checking route plan against hazards, manoeuvring limitation (e.g., rate of turn (ROT)), drafting and refining the route plan against meteorological information [8].
- Collision Avoidance: “*The navigational task of detecting and plotting other ships and objects to avoid collisions*” [9].
- Navigation Control Data: “*Task that provides information for the manual and automatic control of the ship’s movement on a task station*” [9].
- Navigational Status and Data Display: The task that displays data for the manual and automatic control of the ship’s primary movement [8].
- Alert Management: “*Concept for the harmonized regulation of the monitoring, handling, distribution and presentation of alerts on the bridge*” [9].

### 2.2. MITRE ATT&CK Framework

The ATT&CK framework (which stands for Adversarial Tactics, Techniques, and Common Knowledge) has been developed by MITRE since 2013 [10]. It is a globally accessible database including attack tactics, techniques, and mitigation measures for the matrices of enterprise, mobile, and industrial control systems (ICS). The *Enterprise Matrix* covers offensive information (i.e., tactics and techniques) for information technology (IT) networks and cloud services, such as operating systems (i.e., Windows, Linux, and macOS), network components, Office 365, and Google Workspace [11,12]. The *Mobile Matrix* includes offensive knowledge for iOS and Android platforms [13]. The *ICS Matrix* provides offensive information for the ICS [14]. The *Tactics* represents the attack objective, such as initial access, credential access, and lateral movement [15]. *Techniques* expresses methods to achieve an attack objective [16]. The ATT&CK framework also provides mitigation measures to avoid a technique from being successfully executed [17]. Moreover, malware and tools which can be used for malicious purposes are described under the name of *Software* [18]. Another important dimension of ATT&CK is to offer cyber-threat intelligence. *Groups* refers to adversary actor and give techniques implemented and software used by them for an attack in the past [19]. *Data Sources* provides information about various subjects and notions [20].

### 2.3. Literature Review

In the literature, papers implementing various methods have assessed the cyber risks of autonomous ships and conventional ships. Kavallieratos and Katsikas [21] implemented STRIDE and DREAD methods for the cyber risk assessment of several systems on the autonomous ship, such as a collision avoidance system, RAdio Detecting Additionally Ranging (RADAR), closed-circuit television (CCTV), Voyage Data Recorder (VDR), cargo management system, and autopilot. Kavallieratos et al. in [22] also implemented STRIDE for an Automatic Identification System (AIS), engine automation system, bridge automation system, shore control center, engine efficiency system, navigation systems, autonomous ship controller, and so on. Tusher et al. [23] have a cyber risk assessment work for autonomous ships, as well. In their study, the Bayesian best–worst method was implemented, and the authors revealed navigation systems as the most vulnerable element in the context of future autonomous shipping operations. Shang et al. [24] implemented the combination of fuzzy set theory and the Attack Tree method to assess cyber risks of the control system for a gas turbine onboard ship. Oruc [25] also combined fuzzy set theory with another risk assessment method, Fine–Kinney. In the study, 31 cyber risks in the bridge, engine room, and cargo control room onboard a tanker were assessed. Moreover, the efficiency of proposed mitigation measures is shown by implementing the method a second time after taking precautions. Kessler et al. [26] focused on 16 different cyber risks of an AIS. Their study reveals that the disruption of an individual AIS message is more crucial than being unusable of an entire AIS. Svilicic et al. [27] also performed a risk assessment for a specific component. The authors made a cyber risk assessment for the ECDIS on a training vessel by using a vulnerability scanner, named Nessus Professional, and interviewing the ship crew. Several cyber threats were determined regarding the operating system, procedures, awareness, and so on. iTrust published a guideline [28] to uncover cyber risks of operational technology (OT) systems on conventional vessels, including navigation, machinery, communication, and cargo management systems. The traditional risk calculation formula (risk = severity × likelihood) was implemented to assess cyber risks. The study also proposes actionable mitigation measures. You et al. [29] focused on risk assessment methods in other fields and discussed their adaptation to the maritime industry. According to the study, Attack Tree, simulations, and models can be implemented for the cyber risk assessment of marine systems.

Novel methods other than well-established methods are also available in the literature for cyber risks onboard ships. Tam and Jones [30] developed a model-based framework for maritime cyber-risk assessment, entitled Maritime Cyber-Risk Assessment (MaCRA). The authors also implemented the method to assess the cyber risks of three autonomous ship projects in a separate paper [31]. Bolbot et al. [32] proposed a novel method, named CYber-Risk Assessment for Marine Systems (CYRA-MS), by considering the Preliminary Hazard Analysis (CPHA) method to assess cyber risks of ship systems. The authors implemented the method on navigation and propulsion control systems of a fully autonomous inland ship. Meland et al. [33] offered an alternative method for cyber risk assessment. The likelihood of a threat in new design systems is a challenge. The authors propose the threat likelihood approach to support security decision-making for new design systems in particular. Their method is the combination of current concepts, techniques, expert judgements, and domain-specific information.

The ISO 31000 is the root standard and comprises principles, a framework, and a process for risk management [34]. The standard offers a common approach for any size of organization to manage any kind of risk, including the decision-making process [34]. The ISO/TR 31004 explains the effective implementation of ISO 31000 in detail [35]. The IEC 31010 clarifies the selection and application of risk assessment techniques in different situations [36]. The ISO 27000 is another root standard and gives a general approach to information security management systems [37]. The IEC 63154 identifies requirements, test methods, and required test results against cyber incidents for shipborne navigational aids, radio, and navigational equipment [38]. The Formal Safety Assessment (FSA) [39] published by the IMO is a systematic methodology to enhance safety in the maritime industry, including the protection of human life, health, the marine environment and property by using risk analysis. The circular describes the notions, methods, and control measures for a risk assessment. The FSA gives an overall knowledge for a risk assessment in the maritime industry but is not designed specifically for cyber risk assessment.

As mentioned before, IMO issued a regulation for the assessment of cyber risks [4]. After this regulation particularly, several guidelines were published by class societies and other IMO-recognized organizations to support the maritime industry against cyber risks [40,41,42]. The Guidelines on Maritime Cyber Risk Management [42] jointly developed by several industry associations are officially recommended by the IMO [3,43]. The guidelines provide detailed explanations in different dimensions of cyber security, such as cyber threats, risk management, technical and procedural protection measures, and contingency plans, including response and recovery procedures for the maritime industry.

Various comparisons among high-level models, such as the ATT&CK framework, Cyber Kill Chain, OWASP top 10, STRIDE, and the Diamond Model exist [44,45,46,47]. Even though such models are effective in understanding processes and adversary goals, models other than the ATT&CK framework are not useful for explaining the impact of an action to another [48]. Furthermore, the ATT&CK framework depicts correlations of actions with data sources, defenses, configurations, and other countermeasures used for the security of a platform [48].

Even though ATT&CK framework is not a risk assessment method, papers using ATT&CK framework are available for different purposes in other domains, such as risk assessment and risk identification [49,50]. In our study, we reveal that the ATT&CK framework can be used for cyber risk assessment of ship systems as well. Moreover, in the literature, any risk assessment focusing on an INS was not found. Papers in the literature typically assessed the cyber risks of a few components. In our study, we assessed cyber risks for 25 marine components.

## 3. The Extended Methodology and Implementation

Our methodology was derived from the [51] to specialize cyber risks of vessels. The method is based on a Failure Mode Effects and Criticality Analysis (FMECA) and the MITRE ATT&CK framework. The core advantage of the original method is to reduce the need for expert judgement. Thus, the impact of bias in a risk assessment reduces. Moreover, the method is comprehensive and semi-automated. Mitigation measures for cyber risks are included. Our adapted methodology for marine systems is performed as follows:

1. Components are specified and classified.
2. Functions of components and data flow among components are identified.
3. The failure modes for components are determined.
4. Failure modes are mapped with consequences and impacts.
5. Estimation criteria for criticalities are identified.
6. Detection methods and existing controls are identified.
7. The impact scores of components are identified.
8. Risk scores are calculated and risk levels are identified.

### 3.1. Component Specification and Classification

Our methodology starts with the specification and classification of marine components. We implemented our risk assessment methodology on an INS in this study. An INS consists of various marine components. We found 25 components for an INS in our previous study [52]. Such components were classified by IMO and method definitions, respectively. The method definitions for the classification of components are given in Table 1 (e.g., IT, OT, Wireless). Classification by the method definitions is required for the risk assessment process. However, the classification by the IMO definitions is given to provide an additional contribution and to understand the differences between classifications in Table 2.

According to the IMO, components are divided into two groups, such as information technology (IT) and operational technology (OT), and the difference between IT and OT systems is defined as “*Information technology systems may be thought of as focusing on the use of data as information*”, and *“Operational technology systems may be thought of as focusing on the use of data to control or monitor physical processes*” [3]. Moreover, the IMO-recommended document, Guidelines on Maritime Cyber Risk Management, expresses that “*IT covers the spectrum of technologies for data storing and processing, including software, hardware, and communication technologies*”, and “*OT includes hardware and software that directly monitors/controls physical devices and processes, typically on board*.” [42]. Various maritime cyber security-related guidelines were reviewed to find a reliable classification list for marine components by such definitions. However, some marine components, such as ECDIS, RADAR, gyro compass, AIS, global positioning system (GPS), and Bridge Navigational Watch Alarm System (BNWAS) are classified as OT by several organizations [40,41,42]. A full list for INS components has not been found. We classified INS components considering IMO definitions as shown in Table 2. The table also includes columns for *Type*, *Platform*, and *Technology*. The *Type* of the components, such as sensors, Human–Machine Interface (HMI), control server, and engineering workstation was determined. For switches (e.g., the Rudder pump selector switch), we ignored the *Type*. If a component needs an operating system to run, it was stated in the *Platform*. The *Technology* refers to attached technologies such as Wi-Fi, cellular, and Bluetooth.

### 3.2. Functions of Components and Data Flow among Components

In the second step of the method, the functions of the components and data flow among the components are investigated. Such knowledge for an INS was taken from our previous article, as shown in Table 3 [52]. Data flow in the table was identified as per the minimum requirements of the IMO. However, additional connections among the components are allowed.

**Figure 1:**
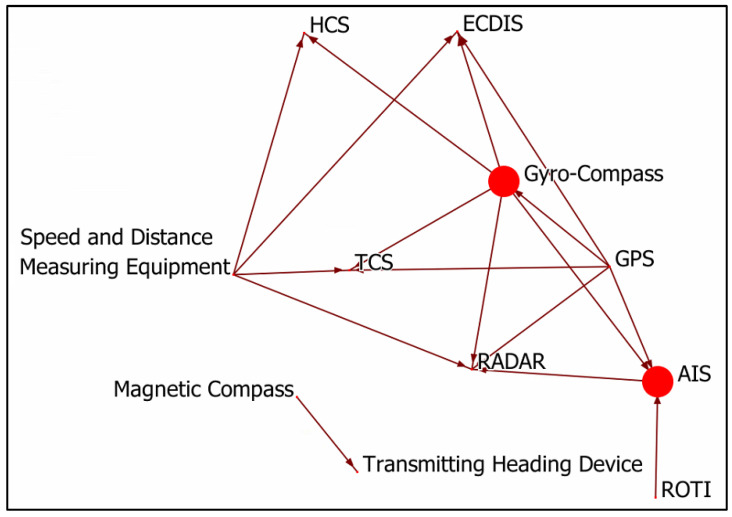
Graph on dependency of INS components.

The ORA is a network tool to analyze, visualize, fuse, and forecast behaviour given network data [53]. Vulnerabilities, model network changes over time, and key players can be identified and formatted reports can be received [54]. Moreover, it consists of tools for optimizing a network’s design structure [54]. In our study, the ORA was employed to calculate various centrality metrics, such as authority, betweenness, and in-degree. Then, the dependency graph was drawn, based on Table 3. The dependency graph among the components is illustrated in Figure 1. In this graph, the nodes represent the investigated component in the INS while the edges represent the identified data flow between components. For instance, as stated in Table 3, the GPS component sends positioning information to the AIS component. This dictates the definition of an edge originating from the GPS component to the AIS component. Additionally, the node size highlights the importance of the node in the network, which is inferred from the nodes’ centrality measurements.

### 3.3. Identifying Failure Modes

The literature was reviewed to understand occurred cyber incidents onboard ships and threats and vulnerabilities of the marine components found in research activities. Moreover, the guidelines of products were reviewed to understand potential failures of components. Component damages and installation mistakes were ignored. In this way, potential failures caused by a cyber attack were determined. Then, failure modes were determined. In this study, failure mode refers to *Tactics* [15] in the ATT&CK framework and is given in three categories, such as Mobile, Enterprise, and ICS. Samples of findings are represented in Table A2.

Then, the possible causes of failure modes or attack techniques were identified and their likelihood was estimated. The identification was performed component-by-component by detecting relationships between components and techniques based on matching attributes. The ATT&CK framework provides attributes of relevant asset types and platforms for each technique. This allows for the identification of the relevant techniques for each component in the system based on the system category. For instance, “Alarm Suppression” is an attack technique against several categories of ICS components such as “RTU”; therefore, “Alarm Suppression” technique would be assigned among the threats identified for any system component that can be categorized as an “RTU”. Afterwards, the likelihood of each technique was calculated based on the exploitability score in the Common Vulnerability Scoring System (CVSS). This entails the estimation of the techniques likelihood based on a Bayesian network of four elements, namely, Attack Complexity (*AC*), Privilege Required (*PR*), Attack Vector (*AV*) and User Interaction (*UI*) using Equation (1):

(1) $$Likelihood_{T}=8.22\times AV\times AC\times PR\times UI\;\left(T:\;Technique\right)$$

Equation (1) is adapted from the CVSS for calculating the exploitability score to maintain alignment with a widely recognized approach for calculating likelihood [55]. The *AC*, *PR*, *AV*, and *UI* information was system-independent and encoded in a Threat Description Table (TDT), and was adopted for all the list of techniques from the original methodology [51].

### 3.4. Mapping Failure Modes with Consequences and Impacts

The consequence is an outcome of an accident [39]. In the original method, consequences are identified as operational, safety, information, financial, and staging. The IMO recommends assessing environmental risks in the FSA [39]. Moreover, we investigated several risk assessment matrices used in the maritime industry and noticed that reputation consequence is also assessed by tanker operators, in particular. Because of such reasons, we extended the method with reputation and environmental consequences.

*Safety Consequence* depicts the potential to cause harm to persons (e.g., crew and passengers). *Operational Consequence* describes potential disruptions, such as errors in the systems during cargo handling. *Financial Consequence* refers to economic losses such as component damages, or commercial losses (e.g., charter party violations). *Information Consequence* explains possible privacy or/and confidentiality violations, such as hosted and processed data in a component. *Staging Consequence* describes the effect of a failure mode which facilitates the staging of future attacks. *Environmental Consequence* describes the potential to cause harm to the environment (e.g., air and water pollution). Reputation Consequence describes harm to company prestige and business life.

Operational, Information, and Staging consequences were broken into impacts. Three metrics are available for estimating the impact on operational consequence, namely the Overall Operational Impact (OOI), Impact to the Control Functions (I2CF), and Impact to the Monitoring Functions (I2MF). If a failure mode (e.g., manipulation of control) impacts the control, it is estimated using the I2CF. If a failure mode (e.g., loss of view) impacts monitoring, it is estimated using the I2MF. Others are estimated using the OOI metric. Staging was estimated using Overall Component Criticality (OCC) and Outbound Degree Centrality (ODC). The failure modes of persistence, defense evasion, and privilege were estimated using the OCC. Others are estimated using the ODC metric. Three types of metrices exists for the information consequence. These are Data Criticality (DC), Intellectual Property Criticality (IPC), and Location Information Criticality (LIC). DC relates to hosted and processed data in a component (e.g., crew information). IPC relates to the hosting of processes with intellectual value. LIC relates to the location information of a component (e.g., position information of a vessel).

Any components in the context of an INS do not process or host personal and confidential data. One feature of an AIS is to transmit location information frequently. When an AIS is equipped mandatorily, it must be always active at anchor and underway unless the master decides to switch it off due to safety and security concerns [56]. However, this decision should be recorded in the logbook with reasons and reported to authorities [56]. Moreover, Long-Range Identification and Tracking (LRIT) onboard also transmit position information [57]. Because of such regulations, the position information of a vessel can not be confidential. Components of an INS are easily found in the market. Furthermore, component standards are identified by the IMO. This is why intellectual property does not existing for an INS. Because of such reasons, an INS is not subject to information consequences. Failure modes were mapped with other consequences and potential impacts for an INS, as illustrated in Table 4.

### 3.5. Identified Estimation Criteria for Criticalities

The estimation criteria were identified for safety, financial, environmental, and reputational criticalities. We proposed estimation criteria for such criticalities. The scores in the estimation criteria tables were identified between 0 and 1 using their impact degrees. Table 5 was used to estimate the impact of a failure mode on the safety consequence. Table 6 was used to forecast financial criticality. The estimation criteria for environmental criticality are depicted in Table 7. Table 5 and Table 7 were derived from the *Appendix 4*—*Initial Ranking of Accident Scenarios* in the FSA published by the IMO [39].

Because of cyber incidents, the seaworthiness and cargo worthiness of a ship may be lost or the ship might be delayed to its destination port. In such cases, the master may need to inform charterers or maritime regulators, such as the port state, flag state, and class society. This would explicitly damage the reputation of the ship operator. This is why we identified two criteria for reputation criticality, as shown in Table 8.

### 3.6. Identifying Detection Methods and Existing Controls

Technical and procedural mitigation measures for enterprise [17], mobile [58], and ICS [59] matrices are given in the ATT&CK framework. Over 70 mitigation measures were assessed for each component in the context of an INS. In Table 9, samples of mitigation measures for components are illustrated. The number “1” in the table refers to that the mitigation measure can be implemented for the component. On the other hand, “0” in the table denotes that the mitigation measure cannot be implemented for the component.

This table assists in calculating the detectability of techniques that can be addressed by certain mitigation measures. Detectability is a term utilized in the original methodology [51] that refers to the degree of risk reduction due to the availability of risk mitigation measures. The detectability of a technique when targeting a specific component is calculated based on Equation (2):

(2) $$Detectability_{T,C,M}=Coverage_{M,C}\times Efficiency_{T,M}\;\left(T:\;Technique,\;C:\;Component,\;M:\;Mitigation\;measure\right)$$

The coverage of a mitigation measure (*M*) for a component (*C*) is referred to in Table 9 while the efficiency of a mitigation measure (*M*) in reducing the risk of a technique (*T*) is estimated for each mitigation measure. In this paper, for simplicity, the efficiency was assumed as 0.5 for all mitigation measures due to the lack of such estimation.

### 3.7. Identifying Impact Scores of Components

Information impacts (i.e., IPC, DC, LIC) were not available for an INS as mentioned in Section 3.4. During the literature review, no incidents harming humans or the environment were found to be caused by cyber attacks against a vessel. This is why safety criticality and environmental criticality were assumed to be in the category *None*—*No injury or insufficient data*. Various aspects affect financial losses, including violation of the charter party agreement, daily operational expenses, repair costs, and so on. It is difficult to estimate a potential loss; however, it is highly possible for this to be over $10,000. This is why financial criticality was assumed as *Significant*—*$10,001*—*$100,000*. The loss of various components may cause the delay of a vessel or the need to inform maritime regulators, such as AIS, GPS, or RADAR. Such components are assumed as *Significant* for reputational criticality. The OOI is the normalized average of all centrality metrics of a component calculated using ORA. ODC denotes the out-degree centrality of a component calculated using ORA. OCC is the overall component criticality, which is calculated using an equation in [51]. It is basically the average of all impacts (e.g., safety, financial, and information). All such assumptions and calculations are represented in Table 10.

### 3.8. Calculating Risk Scores and Identifying Risk Levels

The last element that is required for calculating the risk is the impact of techniques targeting components. This is achieved by utilizing the information in Table 4, Table 10 and Table A2. Table A2 specifies the relevant failure modes for a component. Table 4 specifies the metric to be utilized for estimating the impact of failure mode, and Table 10 specifies the quantification of the impact for each impact element. The final value of the impact of failure mode (*F*) for component (*C*) was calculated using Equation (3):

(3) $$Impact_{F,C}=\left(SF_{F}\times SC_{C}\right)+\left(FF_{F}\times FC_{C}\right)+\left(ICF_{F}\times IC_{C}\right)+\left(OF_{F}\times OC_{C}\right)+\left(StF_{F}\times StC_{c}\right)$$

where *SF_F_*, *FF_F_*, *ICF_F_*, *OF_F_*, and *StF_F_* are the weighting factors for safety, financial, information criticality, operational, and staging impact elements. These factors are expected to be driven from the risk management strategy to prioritize certain impact elements (e.g., safety). In this paper, all impact elements are treated equally, rendering all the factors to be (=1). Additionally, *SC_C_*, *FC_C_*, *IC_C_*, *OC_C_*, and *StC_C_* are the quantification of the impact element for the component (*C*) based on which metric specified for the failure (in Table 4) and the value of that metric (in Table 10). Afterwards, a risk priority number (*RPN*) can be calculated for each identified technique, leading to a failure mode for each component based on Equation (4):

(4) $$RPN_{T,C}=Likelihood_{T}\times Impact_{F,C}\times Detectability_{T,M}\;T:\;Technique,\;C:\;Component,\;F:\;Failure,\;M:\;Mitigation\;measure$$

The likelihood quantification is derived from Equation (1), the impact is derived from Equation (3), and the detectability is derived from Equation (2).

Our findings were prepared in Excel tables as described in [51]. Then, risk scores were calculated by the script, which was specifically coded for the methodology. In the original method, the risks are classified for levels of low risk rating (0–4.86), medium risk rating (4.87–9.72), high risk rating (9.73–14.58), and critical risk rating (14.59–19.44). However, in this study, we ignored several consequences, as described in Section 3.7. This is why we re-defined the risk levels by scores. According to our findings, risks are in the range of 0.041624847 and 8.68820705893103. The range was divided into four classes to prioritize the risks, as shown in Table 11.

In this study, cyber risks for 25 components in an INS were investigated. Three components, such as rudder pump selector switch, steering mode selector switch, and steering position selector switch do not include any cyber risks. A total of 1850 risks belonging to the rest of 22 components were found. Our results classified 1805 risks as low, 32 as medium, 9 as high, and 4 as critical. Risk numbers for each component and risk levels by the original method and our study definitions are represented in Table 12. Medium, high, and critical risks are listed in Appendix A.

Nine high risks were related to AIS, ECDIS, MFD, NAVTEX, and RADAR. RADAR solitarily included four of nine high risks. In total, 1502 risks of 1850 total were related to ECDIS (499 risks), MFD (499 risks), and RADAR (504 risks). The remaining risks related to 19 components. Moreover, four critical risks related to ECDIS and RADAR. A total of 1497 risks for enterprise, 342 risks for ICS, and 11 risks related to the mobile matrix; in total, 443 different techniques led to 1850 risks, 13 of which might compromise over 9 risks as represented in Table 13.

## 4. Conclusions

We proposed a derived method to assess the cyber risks of ships. The original method was developed to assess cyber risks of cyber-physical systems by following the FMECA and MITRE ATT&CK framework. We adapted the method for marine systems in particular. Then, we implemented the method to assess the cyber risks of an INS, and 1850 risks related to 22 components were found. Any risks for three components (i.e., switches) were not available. The risks were classified as 1805 low, 32 medium, 9 high, and 4 critical.

The high and critical risks reflect adversarial objectives to cause an impact on the INS functions. This includes a wide range of threats, such as several variations of denial of service attacks, denial of the processing of sensor data, jamming attacks, and hijacking the resources of sensitive components.

The ECDIS, MFD, and RADAR are the only components that need an operating system to run. According to our results, the operating system increases the cyber threats to and vulnerabilities of a component dramatically. Other components underlying the operating system onboard, such as the ballast water management system and any transfer systems (e.g., bunker), would involve many cyber risks similar to the ECDIS, MFD, and RADAR.

In the original method, consequences are identified as operational, safety, information, financial, and staging. Because of the industry’s necessities, we also took into environmental and reputational consequences. The impact estimation criteria for each consequence were adapted by considering FSA. Information consequence was not available for an INS. Safety and environmental consequences could be possible; however, any marine casualty (e.g., collision, injury, and explosion) caused by cyber incidents does not exist in the literature to date. This is why safety and environmental criticalities could be assumed or ignored. We decided to ignore both. For this reason, we also re-classified risk levels by risk scores. If we had not re-classified the risk levels, the risks would have been underestimated. Once the literature is enriched, other consequences must be considered as well.

The IMO only defines the minimum standards for marine components. Each manufacturer is usually free in various aspects, such as product design, working principle, software, hardware, and operating system. Features, more than requirements, may be attached to products by makers to create added value. This is why failure modes and mitigation measures could be changeable by products. In this study, an implementation of our proposed method is represented and the risk assessment was performed for a typical INS. However, the method is convenient to be implemented in the cyber risk assessment of marine systems other than INS. In further studies, cyber risks of other systems in the bridge, such as safety, security, and communication systems, can be assessed. Moreover, cyber risks of equipment in other locations, such as the engine room and cargo control room, may be assessed.

Our study is based on several assumptions, as many risk assessments were conducted. A few records of cyber incidents and experimental studies against marine systems are available in the literature. This is why we also investigated troubleshooting sections of product brochures to assume the impact of a potential attack. The mapping of failure modes and their consequences are subjective and might change under expert judgement. Financial criticality was considered as significant (USD 10,001–100,000). However, commercial losses (e.g., cargo claims, charter party violations, and loss of potential charterer) and costs for components, service, mooring and so on could directly affect the financial losses of a cyber incident. This is why financial impact is based on assumptions, as well. Despite several assumptions, the method is comprehensive and detailed. It can be perfectly implemented to assess the cyber risks of well-defined marine systems under a specific scenario.

The study offers two classifications for components of an INS. The IMO classifies the components as IT and OT. However, our method can classify IT, OT, wireless, and combinations of these. Our method and IMO differently define IT and OT notions. For the risk assessment method, IMO definitions are not required. Given that any complete list could not be found in the literature, component classification for an INS by the IMO definition was also given in our study as an additional contribution.

## Figures and Tables

**Table 1:** Component classification by method [51].

Classification	Description
IT	Components that are hosted on a traditional IT system such as multipurpose computers or network devices.
OT	Components that are involved in monitoring and controlling functions.
Wireless	Components that are connected to a mobile network or communicate with an external infrastructure, such as Aids to Navigation, to acquire location-related information in the maritime domain.
IT/OT	Dual-homed components that are hosted on a traditional IT system and are involved in monitoring and controlling functions.
IT/OT/Wireless	Components that are classified as IT/OT and are connected to a mobile network or communicate with an external infrastructure.

**Table 2:** Components and classification of components.

Component	Classification		Type	Platform	Technology
	IMO	Method			
AIS	OT	IT, OT, Wireless	Sensor		radio, GPS
Anemometer	OT	IT, OT	Sensor		
BNWAS	OT	IT, OT	Sensor		
Central Alert Management HMI	OT	IT, OT	HMI		
Controls for main engine	OT	OT	Control Server		
Controls for main rudder	OT	OT	Control Server		
Controls for thruster	OT	OT	Control Server		
ECDIS	OT	IT, OT	Engineering workstation	OS	
Echo Sounder	OT	IT, OT	Sensor		
GPS	OT	IT, OT, Wireless	Sensor		GPS
Gyro-Compass	OT	IT, OT	Sensor		
Heading Control System (HCS)	OT	IT, OT	Control Server		
Indicators	OT, IT	IT	HMI		
Magnetic Compass	OT	IT, OT	Sensor		
Multi Function Display (MFD)	OT	IT, OT	Engineering workstation	OS	
Navigational Telex (NAVTEX)	OT	IT, OT, Wireless	Sensor		radio
RADAR	OT	IT, OT	Sensor	OS	radio
ROTI	OT	IT, OT	Sensor		
Rudder pump selector switch	OT	OT	N/A		
Sound reception system	OT	IT, OT	Sensor		
Speed and Distance Measuring Equipment (SDME)	OT	IT, OT	Sensor		
Steering mode selector switch	OT	OT	N/A		
Steering position selector switch	OT	OT	N/A		
Track Control System (TCS)	OT	IT, OT	Control Server		
Transmitting Heading Device (THD)	OT	IT, OT	Sensor		

**Table 3:** Functions of components and data flow [52].

Component	Function	Data Flow
AIS	identifying ships, assisting in target tracking, assisting in search and rescue operation, information exchange, providing additional information to assist situation awareness	Sends to: RADAR
Anemometer	detecting and indicating wind speed and direction	N/A
BNWAS	monitoring bridge activity, detecting operator disability and then alerting automatically	N/A
Central Alert Management HMI	reporting abnormal situation which requires an attention	Receives from: sensors connected
Controls for main engine	Control buttons or levers of the main engine for different purposes such as rpm, load, emergency stop button, sailing mode selection button, and so on	N/A
Controls for main rudder	commanding the rudder angel, activating the override mode	N/A
Controls for thruster	commanding the thrusters such as starting, stopping, load/stage, etc.	N/A
ECDIS	offering the functions of route planning, route monitoring and positioning for officers in ECDIS instead of paper charts	Receives from: GPS, gyro compass, SDME. If the ships are not equipped with gyro compass, ECDIS receives data from the transmitting heading device
Echo Sounder	measuring the depth of water under the ship, and presenting graphically	N/A
GPS	providing space-based positioning, velocity and time system	Sends to: AIS, RADAR, ECDIS, HCS, TCS, Gyro compass
Gyro-Compass	determining the direction of the ship’s head in relation to geographic (true) north	Sends to: AIS, RADAR, ECDIS, HCS, TCS Receives from: GPS
HCS	keeping the vessel in preset heading by using heading information	Receives from: Gyro compass or Transmitting Heading Device. Moreover, GPS or SDME
Indicators	shows data or status information received from sensor	Receives from: Sensors connected.
Magnetic Compass	determining and displaying the ship’s heading without any power supply	Sends to: THD
MFD	A display unit presents information from more than a single function of the INS	depends on connected equipment
NAVTEX	receiving and automatically printing or displaying Maritime Safety Information (MSI)	N/A
RADAR	indication, in relation to own ship, of the position of other surface craft, obstructions and hazards, navigation objects and shorelines	Receives from: AIS, GPS, SDME Moreover, Gyro compass or Transmitting Heading Device
ROTI	indicating rates of turn to starboard and to port of the ship to which it is fitted	Sends to: AIS
Rudder pump selector switch	selection of primary and secondary (emergency) hydraulic or electrohydraulic pumps for rudder direction	N/A
Sound reception system	offers the OOW who can hear and determine the direction of the sound signals of the vessels nearby	N/A
SDME	measuring and indicating speed and distance of the vessel	Sends to: HCS, RADAR, ECDIS, TCS
Steering mode selector switch	selection of steering modes, such as “Auto”, “Non-Follow Up”, or “Follow Up”.	N/A
Steering position selector switch	determining the active steering workstation (i.e., port wing, starboard wing or center)	N/A
TCS	Track control system keeps the vessel on a pre-planned track over ground by using position, heading and speed information of the vessel	Receives from: GPS, SDME, Gyro compass
Transmitting Heading Device	indicating ship’s true heading by means of magnetic compass	Receives from: magnetic compass Sends to: AIS, HCS, TCS, ECDIS, RADAR

**Table 4:** Mapping failure modes, consequences, and impacts.

Matrices	Failure Modes	Consequences						
		Operational	Reputation	Environmental	Safety	Information	Financial	Staging
Mobile	Network Denial of Service	I2MF		EC	SC			
	impact	I2MF		EC	SC			
IT	collection							ODC
	credential access		RC					ODC
	data encrypted for impact	OOI	RC	EC	SC		FC	
	data manipulation	OOI	RC	EC	SC		FC	
	discovery							ODC
	execution	OOI	RC	EC	SC		FC	ODC
	exfiltration							ODC
	firmware corruption	OOI		EC	SC		FC	
	initial access							ODC
	lateral movement							ODC
	system shutdown/reboot	OOI		EC	SC		FC	
ICS	collection							ODC
	discovery							ODC
	execution	OOI	RC	EC	SC		FC	ODC
	initial access							ODC
	lateral movement							ODC
	loss of availability	OOI	RC	EC	SC		FC	ODC
	loss of control	I2CF	RC	EC	SC		FC	
	loss of safety	OOI	RC	EC	SC		FC	
	loss of view	I2MF	RC	EC	SC		FC	ODC
	manipulation of control	I2CF	RC	EC	SC		FC	
	manipulation of view	I2MF	RC	EC	SC		FC	ODC

SC: Safety criticality, FC: financial criticality, EC: environmental criticality, RC: reputational criticality.

**Table 5:** Estimation criteria for safety criticality.

Safety Criticality	Description	Score
None	No injury or insufficient data	0
Minor	Single or minor injuries	0.25
Significant	Multiple or severe injuries	0.50
Severe	Single fatality or multiple severe injuries	0.75
Catastrophic	Multiple fatalities	1

**Table 6:** Estimation criteria for financial criticality.

Financial Criticality	Description (USD)	Score
None	No financial loss or insufficient data	0
Minor	1–10,000	0.25
Significant	10,001–100,000	0.50
Severe	100,001–1,000,000	0.75
Catastrophic	Financial loss > 1,000,000	1

**Table 7:** Estimation criteria for environmental criticality.

Environ. Criticality	Description	Score
None	No environmental damage or insufficient data	0.00
Minor	Oil spill size < 1 tonne	0.20
Significant	Oil spill size between 1–10 tonnes	0.40
Severe	Oil spill size between 11–100 tonnes	0.60
Catastrophic	Oil spill size between 101–1000 tonnes	0.80
Extreme	Oil spill size > 1000 tonnes	1

**Table 8:** Estimation criteria for reputational criticality.

Reputation Critical.	Description	Score
None	None	0
Significant	Notification requirement to third parties	1

**Table 9:** Samples for risk-mitigation measures.

Component	Samples for Mitigation Measures														
	Account Use Policies	Active Directory Configuration	Antivirus/Antimalware	Application Developer Guidance	Application Isolation and Sandboxing	Audit	Behavior Prevention on Endpoint	Boot Integrity	Code Signing	Credential Access Protection	Data Backup	Data Loss Prevention	Disable or Remove Feature or Program	Do Not Mitigate	Encrypt Sensitive Information
AIS	0	0	0	0	0	1	0	1	0	0	0	0	0	0	0
Anemometer	0	0	0	0	0	1	0	0	0	0	0	0	0	0	0
BNWAS	0	0	0	0	0	1	0	0	0	0	0	0	0	0	0
Central Alert Management HMI	0	0	0	0	0	1	0	1	0	0	0	0	0	0	0
Controls for M/E	0	0	0	0	0	1	0	0	0	0	0	0	0	0	0
Controls for main rudder	0	0	0	0	0	1	0	0	0	0	0	0	0	0	0
Controls for thruster	0	0	0	0	0	1	0	0	0	0	0	0	0	0	0
ECDIS	0	1	1	0	0	1	1	1	0	1	1	0	1	0	1
Echo Sounder	0	0	0	0	0	1	0	1	0	0	0	0	0	0	0
GPS	0	0	0	0	0	1	0	1	0	0	0	0	0	0	0
Gyro-Compass	0	0	0	0	0	1	0	0	0	0	0	0	0	0	0
HCS	0	0	0	0	0	1	0	1	0	0	0	0	0	0	0
Indicators	0	0	0	0	0	1	0	0	0	0	0	0	0	0	0
Magnetic Compass	0	0	0	0	0	1	0	0	0	0	0	0	0	0	0
MFD	0	1	1	0	0	1	1	1	0	1	1	0	1	0	1
NAVTEX	0	0	0	0	0	1	0	1	0	0	0	0	0	0	0
RADAR	0	1	1	0	0	1	1	1	0	1	1	0	1	0	1
ROTI	0	0	0	0	0	1	0	0	0	0	0	0	0	0	0
Rudder pump selector switch	0	0	0	0	0	1	0	0	0	0	0	0	0	0	0
Sound reception system	0	0	0	0	0	1	0	0	0	0	0	0	0	0	0
SDME	0	0	0	0	0	1	0	0	0	0	0	0	0	0	0
Steering mode selector switch	0	0	0	0	0	1	0	0	0	0	0	0	0	0	0
Steering position selector switch	0	0	0	0	0	1	0	0	0	0	0	0	0	0	0
TCS	0	0	0	0	0	1	0	1	0	0	0	0	0	0	0
Transmitting Heading Device	0	0	0	0	0	1	0	0	0	0	0	0	0	0	0

**Table 10:** Component criticality score table.

Component	Information			SC	EC	FC	RC	OOI	Staging	
	IPC	DC	LIC						ODC	OCC
AIS	0	0	0	0	0	0.5	1	0.872174439	0.042	0.402362407
Anemometer	0	0	0	0	0	0.5	0	0	0	0.083333333
BNWAS	0	0	0	0	0	0.5	0	0	0	0.083333333
Central Alert Manageme. HMI	0	0	0	0	0	0.5	0	0	0	0.083333333
Controls for M/E	0	0	0	0	0	0.5	1	0	0	0.25
Controls for main rudder	0	0	0	0	0	0.5	1	0	0	0.25
Controls for thruster	0	0	0	0	0	0.5	0	0	0	0.083333333
ECDIS	0	0	0	0	0	0.5	1	0.438221675	0	0.323036946
Echo Sounder	0	0	0	0	0	0.5	0	0	0	0.083333333
GPS	0	0	0	0	0	0.5	1	0.7350904	0.208	0.407181733
Gyro-Compass	0	0	0	0	0	0.5	1	1	0.208	0.284666667
HCS	0	0	0	0	0	0.5	0	0.301782611	0	0.133630435
Indicators	0	0	0	0	0	0.5	0	0	0	0.083333333
Magnetic Compass	0	0	0	0	0	0.5	0	0.149697807	0.042	0.115282968
MFD	0	0	0	0	0	0.5	1	0	0	0.25
NAVTEX	0	0	0	0	0	0.5	1	0	0	0.25
RADAR	0	0	0	0	0	0.5	1	0.735171456	0	0.372528576
ROTI	0	0	0	0	0	0.5	0	0.177510045	0.042	0.119918341
Rudder pump selector switch	0	0	0	0	0	0.5	0	0	0	0.083333333
Sound reception system	0	0	0	0	0	0.5	0	0	0	0.083333333
SDME	0	0	0	0	0	0.5	0	0.552742648	0.167	0.203290441
Steering mode selector switch	0	0	0	0	0	0.5	0	0	0	0.083333333
Steering position selector switch	0	0	0	0	0	0.5	0	0	0	0.083333333
TCS	0	0	0	0	0	0.5	0	0.438221675	0	0.156370279
Transmitting Heading Device	0	0	0	0	0	0.5	0	0.156940387	0	0.109490065

**Table 11:** New risk scores with levels.

Range	Level
0.00–2.18	Low
2.19–4.36	Medium
4.37–6.54	High
6.55–8.72	Critical

**Table 12:** Results of risk assessment.

Component	Total Risk	Risk Level (Original)	Risk Level (Study)
AIS	5	5 low	3 low 1 medium 1 high
Anemometer	5	5 low	5 low
BNWAS	5	5 low	5 low
Central Alert Management HMI	41	41 low	41 low
Controls for M/E	40	40 low	35 low 5 medium
Controls for main rudder	40	40 low	35 low 5 medium
Controls for thruster	40	40 low	40 low
ECDIS	499	496 low 3 medium	489 low 7 medium 1 high 2 critical
Echo Sounder	5	5 low	5 low
GPS	5	5 low	4 low 1 medium
Gyro-Compass	5	5 low	5 low
HCS	40	40 low	39 low 1 medium
Indicators	41	41 low	41 low
Magnetic Compass	5	5 low	5 low
MFD	499	497 low 2 medium	492 low 3 medium 2 high
NAVTEX	11	10 low 1 medium	9 low 1 medium 1 high
RADAR	504	501 low 3 medium	492 6 medium 4 high 2 critical
ROTI	5	5 low	5 low
Rudder pump selector switch	0		
Sound reception system	5	5 low	5 low
Speed and Distance Measuring Equipment	5	5 low	5 low
Steering mode selector switch	0		
Steering position selector switch	0		
TCS	40	40 low	38 low 2 medium
Transmitting Heading Device	5	5 low	5 low
**Total**	1850	1841 low 9 medium	1805 low 32 medium 9 high 4 critical

**Table 13:** Techniques compromising over 10 risks.

Matrix	MITRE ID	Techniques	Risk Number
ICS	T0858	Change Operating Mode	24
ICS	T0829	Loss of View	14
ICS	T0832	Manipulation of View	14
ICS	T0849	Masquerading	14
ICS	T0859	Valid Accounts	14
ICS	T0886	Remote Services	14
ICS	T0815	Denial of View	12
Enterprise	T1078	Valid Accounts	12
Enterprise	T1078.001	Valid Accounts: Default Accounts	12
Enterprise	T1078.002	Valid Accounts: Domain Accounts	12
Enterprise	T1078.003	Valid Accounts: Local Accounts	12
ICS	T0822	External Remote Services	10
ICS	T0856	Spoof Reporting Message	10

## Data Availability

The data are available upon request via corresponding author email.

## Appendix A

Medium, high, and critical risks of an INS are given in Table A1.

**Table A1:** Medium, high, and critical risks of an INS.

No.	Component	MITRE ID	Techniques	Risk
1	AIS	T0815	Denial of View	High
2	AIS	T0829	Loss of View	Medium
3	Controls for M/E	T0879	Damage to Property	Medium
4	Controls for M/E	T0809	Data Destruction	Medium
5	Controls for M/E	T0826	Loss of Availability	Medium
6	Controls for M/E	T0828	Loss of Productivity and Revenue	Medium
7	Controls for M/E	T0856	Spoof Reporting Message	Medium
8	Controls for main rudder	T0879	Damage to Property	Medium
9	Controls for main rudder	T0809	Data Destruction	Medium
10	Controls for main rudder	T0826	Loss of Availability	Medium
11	Controls for main rudder	T0828	Loss of Productivity and Revenue	Medium
12	Controls for main rudder	T0856	Spoof Reporting Message	Medium
13	ECDIS	T1498.002	Reflection Amplification	Medium
14	ECDIS	T1499.004	Application or System Exploitation	Medium
15	ECDIS	T1499.003	Application Exhaustion Flood	Medium
16	ECDIS	T1499.002	Service Exhaustion Flood	Medium
17	ECDIS	T1499.001	OS Exhaustion Flood	Medium
18	ECDIS	T1531	Account Access Removal	Medium
19	ECDIS	T1529	System Shutdown/Reboot	Medium
20	ECDIS	T1499	Endpoint Denial of Service	Critical
21	ECDIS	T1498	Network Denial of Service	Critical
22	ECDIS	T1496	Resource Hijacking	High
23	GPS	T0815	Denial of View	Medium
24	HCS	T0826	Loss of Availability	Medium
25	MFD	T1531	Account Access Removal	Medium
26	MFD	T1529	System Shutdown/Reboot	Medium
27	MFD	T1499	Endpoint Denial of Service	High
28	MFD	T1498	Network Denial of Service	High
29	MFD	T1496	Resource Hijacking	Medium
30	NAVTEX	T1464	Network Denial of Service	High
31	NAVTEX	T1463	Manipulate Device Communication	Medium
32	RADAR	T1498.002	Reflection Amplification	High
33	RADAR	T1499.004	Application or System Exploitation	Medium
34	RADAR	T1499.003	Application Exhaustion Flood	Medium
35	RADAR	T1499.002	Service Exhaustion Flood	High
36	RADAR	T1499.001	OS Exhaustion Flood	High
37	RADAR	T1491.001	Internal Defacement	Medium
38	RADAR	T1531	Account Access Removal	Medium
39	RADAR	T1529	System Shutdown/Reboot	Medium
40	RADAR	T1499	Endpoint Denial of Service	Critical
41	RADAR	T1498	Network Denial of Service	Critical
42	RADAR	T1496	Resource Hijacking	High
43	RADAR	T1491	Defacement	Medium
44	TCS	T0809	Data Destruction	Medium
45	TCS	T0826	Loss of Availability	Medium

## Appendix B

Table A2 represents samples of failures, cyber incidents, vulnerabilities and failure modes.

**Table A2:** Samples of failures, incidents and vulnerabilities, and failure modes.

Component	Failure	Occurred Incidents & Discovered Vulnerabilities	Failure Modes		
			Mobile	Enterprise	ICS
AIS	not receiving AIS messages; not transmitting AIS messages; transmitting the wrong AIS messages; displaying invalid AIS information; difference between internal and external GPS data; mismatching heading data.	spoofing; hijacking; availability; tampering.	network denial of service; impact.	data manipulation; firmware corruption; initial access.	loss of availability; loss of control; loss of safety; loss of view; manipulation of control; manipulation of view.
Anemometer	inaccurate wind speed; missing wind speed; inaccurate wind direction; missing wind direction.	N/A		data manipulation.	loss of availability; loss of control; loss of view; manipulation of control; manipulation of view.
BNWAS	not activating/deactivating it in automatic mode; not rising alarm; rising alarm constantly; not working motion detectors if equipped.	N/A		data manipulation; firmware corruption; initial access.	loss of availability; loss of control; loss of safety; loss of view; manipulation of control; manipulation of view.
Central Alert Management HMI	not stopping alert; not rising alert; not keeping alert history; displaying alerts with wrong date/time stamp.	N/A		data manipulation; firmware corruption; initial access.	loss of availability; loss of control; loss of safety; loss of view; manipulation of control; manipulation of view.
Controls for M/E	not changing or RPM; missing or wrong information; not working of command.	N/A			loss of availability; loss of control; loss of safety; loss of view; manipulation of control; manipulation of view.
ECDIS	collapsing the operating system; wrong position of own vessel; not updating ENC/RNC; not receiving/displaying information from connected components; not allowing route planning or monitoring; data manipulation in functions such as past track or planned course.	operating system vulnerabilities; middleware vulnerabilities; manipulation of the ship position.		collection; discovery; execution; exfiltration; initial access; data encrypted for impact; credential access; data manipulation; lateral movement; system shutdown/reboot; defense evasion.	loss of availability; loss of control; loss of safety; loss of view; manipulation of control; manipulation of view.
Echo Sounder	inaccurate depth value; no depth value.	N/A		data manipulation; firmware corruption; initial access.	loss of availability; loss of control; loss of safety; loss of view; manipulation of control; manipulation of view.
GPS	not fixing the position; wrong position; not transmitting the data to other components.	jamming; spoofing.	network denial of service; impact.	data manipulation; firmware corruption; initial access.	loss of availability; loss of control; loss of safety; loss of view; manipulation of control; manipulation of view.
Gyro-Compass	displaying wrong heading information; not receiving GPS messages; not transmitting information to other components.	N/A		data manipulation; firmware corruption; initial access.	loss of availability; loss of control; loss of safety; loss of view; manipulation of control; manipulation of view.
HCS	not receiving NMEA messages from connected components.	N/A		data manipulation; firmware corruption; initial access.	loss of availability; loss of control; loss of safety; loss of view; manipulation of control; manipulation of view.
Indicators	not receiving NMEA messages from connected components.	N/A		data manipulation.	loss of availability; loss of safety; loss of view; manipulation of view.
MFD	not receiving NMEA messages from connected components; collapsing operating system.	operating system vulnerabilities; middleware vulnerabilities.		collection; defense evasion; discovery; execution; exfiltration; initial access; data encrypted for impact; credential Access; data manipulation; lateral movement; system shutdown/reboot.	loss of availability; loss of control; loss of safety; loss of view; manipulation of control; manipulation of view.
NAVTEX	not receiving MSI	N/A	network denial of service; impact.	firmware corruption; initial access.	loss of availability; loss of control; loss of safety; loss of view; manipulation of control; manipulation of view.
